# An ant-coccid mutualism affects the behavior of the parasitoid *Aenasius bambawalei*, but not that of the ghost ant *Tetramorium bicarinatum*

**DOI:** 10.1038/s41598-017-05442-6

**Published:** 2017-07-12

**Authors:** Jun Huang, Peng-Jun Zhang, Juan Zhang, Ya-Yuan Tang

**Affiliations:** 1 Flower Research and Development Centre, Zhejiang Academy of Agricultural Sciences, Hangzhou, 311202 China; 2 Zhejiang Provincial Key Laboratory of Biometrology and Inspection & Quarantine, College of Life Sciences, China Jiliang University, Hangzhou, 310018 China

## Abstract

Mutualisms between honeydew-producing insects and ants change the emission of volatiles from plants, but whether such changes alter the behaviors of ants that tend honeydew-producing insects or wasps that parasitize honeydew-producing insects remain unknown. This study compared the behavioral responses of the ant *Tetramorium bicarinatum* and the parasitoid wasp *Aenasius bambawalei* to odors from cotton plants infested with the mealybug *Phenacoccus solenopsis* or infested with the mealybug and the ant, which tends the mealybug. The ant could not distinguish between the volatiles from plants infested with the mealybug alone and those from plants infested with the mealybug and the ant. Likewise, naïve wasps failed to distinguish between volatiles from the two treatments. In contrast, experienced wasps preferred volatiles from plants infested with the mealybug and the ant. Volatile analysis showed that the amounts of MeSA were increased and those of methyl nicotinate were decreased when plants were infested by the mealybug and the ant rather than when plants were uninfested or were infested by the mealybug alone. Thus, the mutualism between the mealybug and ant changed the volatiles emitted by cotton plants such that the attraction of *A. bambawalei* (but not that of the ant) to the plants was increased.

## Introduction

Mutualisms between honeydew-producing insects (HPIs) (e.g., aphids and mealybugs) and ants are common in nature^1, 2^. Such mutualisms alter the physiology and fitness of the infested plants^2–4^, e.g., the mutualisms affect plant photosynthesis^4^, seed production^5^, pollination^6^, and attack by herbivores^7^. Furthermore, honeydew-collecting ants can increase the survival of HPIs by protecting them from parasitoids^8, 9^.

Based on most previous studies, protection occurs because tending ants directly attack parasitoids and cause parasitoids to take evasive action^8–10^. However, females of some parasitoid species learn and change their behavior, chemistry or morphology in response to ant encounters^8, 11, 12^. Paris *et al*.^13^ determined that ants indirectly change the emission of volatile organic compounds (VOCs) by tending to aphids, and VOCs are important foraging cues for parasitoids of herbivores^14^. Therefore, we hypothesized that mutualisms between ants and HPIs may indirectly affect the behavior of parasitoid females through changes in the emission of VOCs.

Changes in plant VOCs may affect the behavior of parasitoids, of other herbivores, and possibly of tending ants. In ant-plant symbioses, ant queens of some species are attracted to volatile chemical cues emitted by their host plant and use these cues to locate leaf pouches and other specialized structures^15^. In addition, ant recruitment induced in ant-plant systems often resemble rapidly induced chemical responses and are therefore effective against mobile herbivores that can damage plants^16^. Therefore, we hypothesized that the damage caused by the mealybugs will change the volatile compounds and induce rapid ant recruitment or continuous ant patrols of leaves until mealybugs are encountered.

To test these two hypotheses, we chose a mutualism on cotton plants that consisted of the cotton mealybug *Phenacoccus solenopsis* Tinsley (Homoptera: Pseudococcidae) and the tending ghost ant *Tetramorium bicarinatum* (Hymenoptera: Formicidae), which is one of the most widely distributed ant species worldwide^17^. The cotton mealybug is a typical HPI, and is a newly recognized invasive pest in China^18^. Because it produces honeydew, the mealybug is tended by several ant species that collect honeydew as food^4, 10, 19^. *Aenasius bambawalei* (Hymenoptera: Encyrtidae) is a primary parasitoid of the mealybug^20^, and the parasitism rate in the field can exceed 62%^21^. Therefore, this system is suitable for examining interactions between the mealybug, the tending ant, and the parasitoid wasp.

In this paper, we attempted to answer the following questions: (1) Do ant-tended mealybug colonies affect the olfactory response of *A. bambawalei* and does the response of *A. bambawalei* change with experience? (2) Does mealybug infestation (=mealybug injury or damage) induce ant recruitment to plants in the absence of mealybug encounters? and (3) Does mealybug infestation change the volatile compounds emitted by cotton plants? To identify the compounds that changed with the infestation and to identify those linked to ant tending, we sampled volatiles emitted by cotton plants alone and those infested with the mealybug with or without the ghost ant.

## Results

### Wasp attraction to plants with and without mealybugs that were tended or not tended by ants as determined with Y-tube olfactometers (experiment 1)

**Figure 1:**
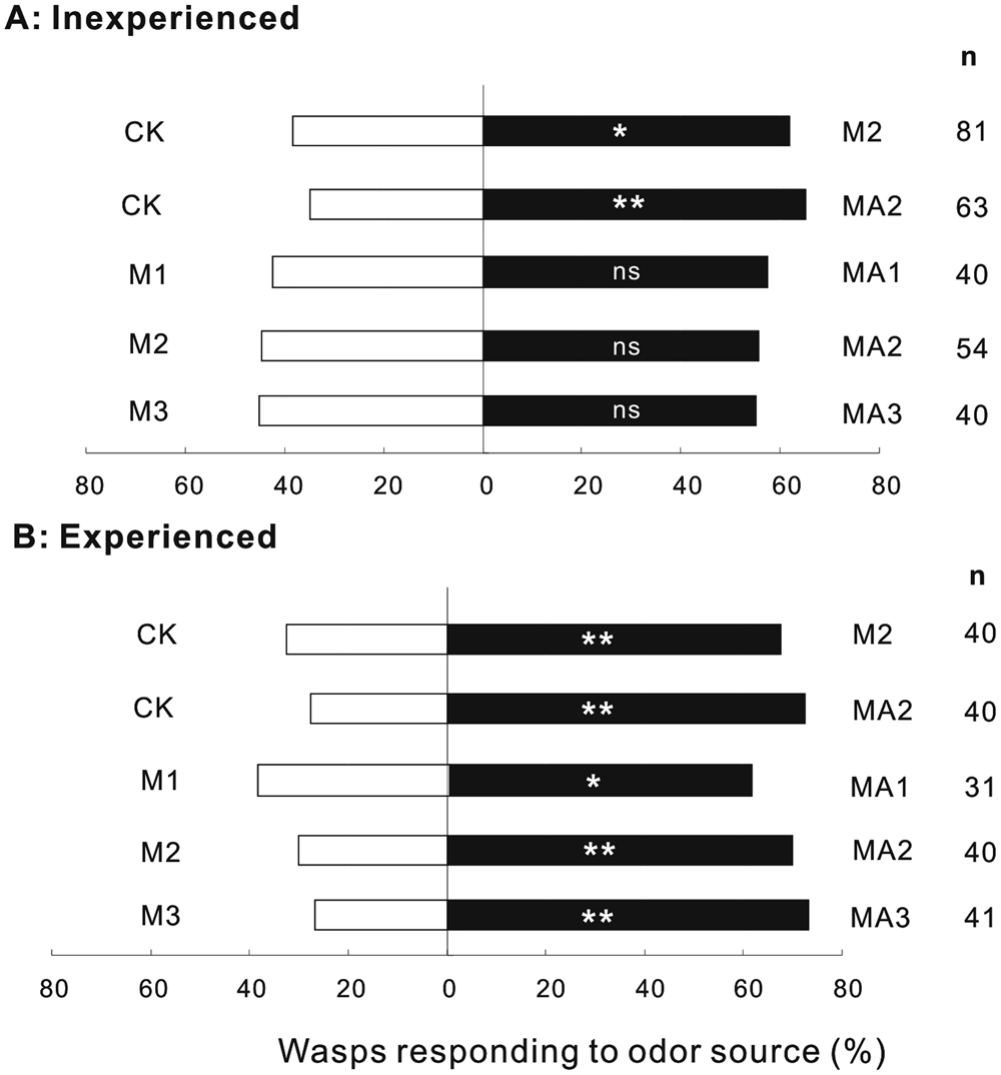
Attraction of experienced and inexperienced *A*. *bambawalei* females to plants with and without mealybugs that were tended or not tended by ants (experiment 1). Control plants were not infested with mealybugs or ants. Other plants were infested with 30, 60, or 90 mealybugs (and therefore had mealybug damage) without ant infestation (treatments M1, M2, and M3, respectively) or with ant infestation (treatments MA1, MA2, and MA3, respectively). Pairs of plants were then used in an olfactometer assay with (**A**) experienced *A. bambawalei* females (previously exposed to plant volatiles) or (**B**) inexperienced *A. bambawalei* females (not previously exposed to plant volatiles). Bars represent the percentages of wasps choosing either of the odor sources (binomial test; * *P* < 0.05, ** *P* < 0.01; ns, not significant).

In experiment 1, control plants were not infested with mealybugs or ants. Other plants were infested with 30, 60, or 90 mealybugs (and therefore had mealybug damage) with or without ant infestation. Pairs of plants were then used in an olfactometer assay with experienced or inexperienced *A. bambawalei* females, i.e., with females that had or had not been exposed to volatiles from plants infested with mealybugs and ants*. Aenasius bambawalei* females significantly preferred cotton plants with mealybugs (*Chi-square test*, inexperienced females-*P* = 0.02, *n* = 81; experienced females-*P* < 0.001, *n* = 40) and those with ant-tended mealybugs (*Chi-square test*, inexperienced females-*P* = 0.003, *n* = 63; experienced females-*P* < 0.001, *n* = 40) over control plants (Fig. 1). When the odor sources from the mealybug and the ant-tended mealybug treatments were compared, inexperienced females showed no preference for any level of mealybug (*Chi-square test*, 30 mealybugs-*P* = 0.13, *n* = 40; 60 mealybugs-*P* = 0.26, *n* = 54; 90 mealybugs-*P* = 0.32, *n* = 40). However, experienced females significantly preferred plants with mealybugs and ants over plants with mealybugs alone (*Chi-square test*, 30 mealybugs-*P* = 0.02, *n* = 31; 60 mealybugs-*P* < 0.001, *n* = 40; 90 mealybugs-*P* < 0.001, *n* = 41).

### Ant responses to mealybug infestation (experiment 2)

**Figure 2:**
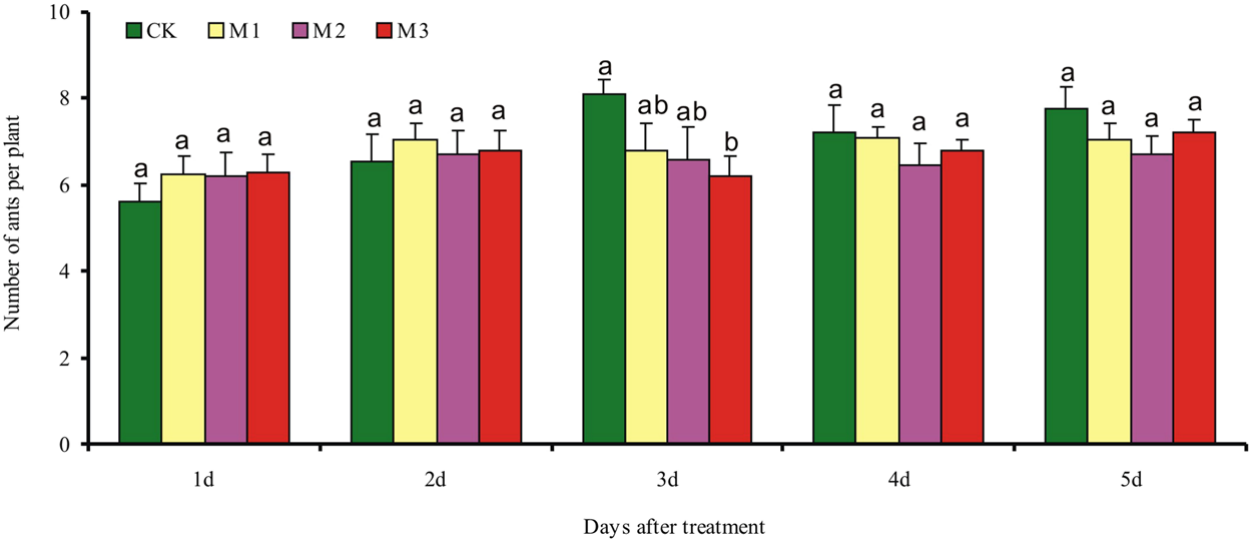
Ant responses to mealybug damage (experiment 2). Plants with one of four levels of mealybugs (CK = 0, M1 = 30, M2 = 60, and M3 = 90 per plant) were placed on a rack sitting in a box with ca. 200 workers plus two queens; the ants in the box could move up the rack to the plants but could not contact the mealybugs because the petioles were coated with petrolatum. The number of ants per plant was then recorded on 5 consecutive days.

When ants were exposed to cotton plants without or with mealybugs (four levels of mealybugs including the control) but were prevented from contacting the mealybugs by placement of white petrolatum on leaf petioles, the number of ants that moved from a box to plants did not differ among treatments or observation days (Fig. 2). The effects were not significant for mealybug level (*F*_3,180_ = 1.07, *P* = 0.362), observation day (*F*_4,180_ = 2.88, *P* = 0.024), or the mealybug level × observation day interaction (*F*_12,180_ = 0.95, *P* = 0.494).

### Ant responses to mechanical damage (experiment 3)

**Figure 3:**
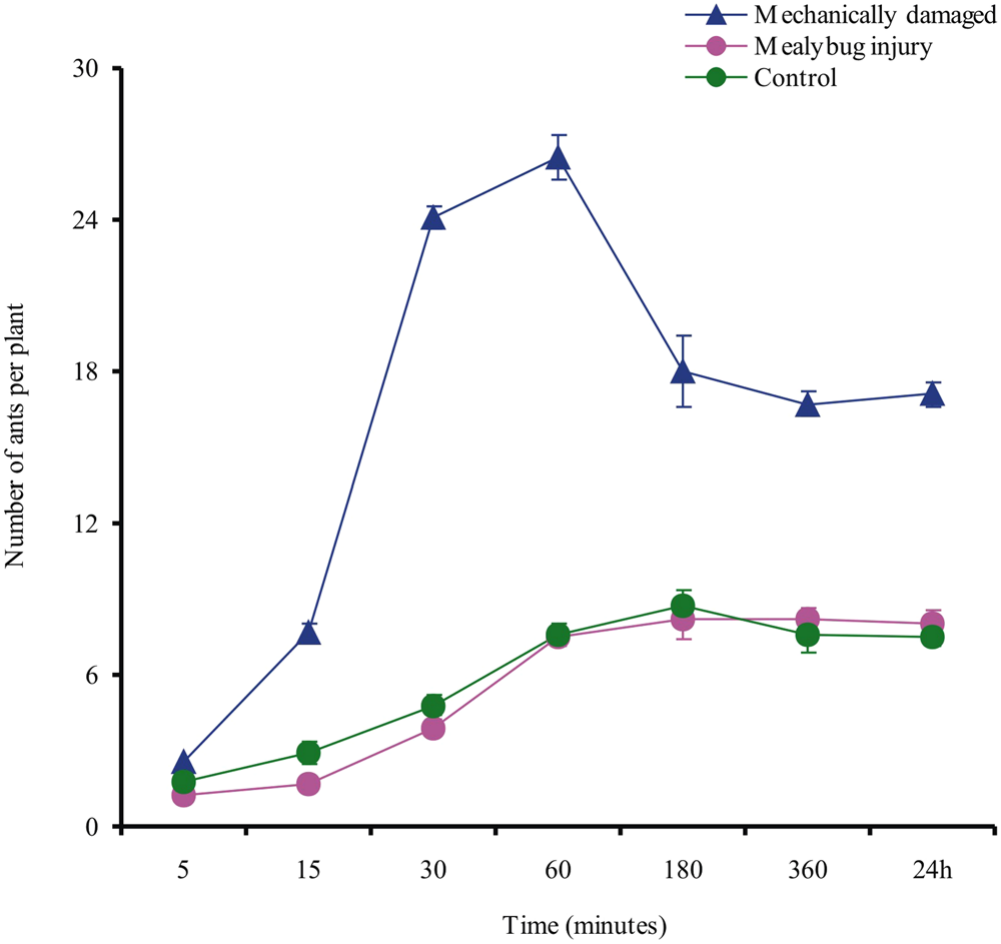
Ant responses to mechanically damaged and mealybug-infested cotton leaves (experiment 3). For mechanical damage, two holes were punched in each of two leaves per plant. For mealybug damage, 60 mealybugs were added per plant. As in experiment 2, the plants were placed on a rack sitting in a box with ca. 200 ants; the ants in the box could move up the rack to the plants but could not contact the leaves because the petioles were coated with petrolatum. Ant numbers per plant were recorded after 5 to 360 min and again after 24 h. Values are the means (±SE) of 10 replications.

**Figure 4:**
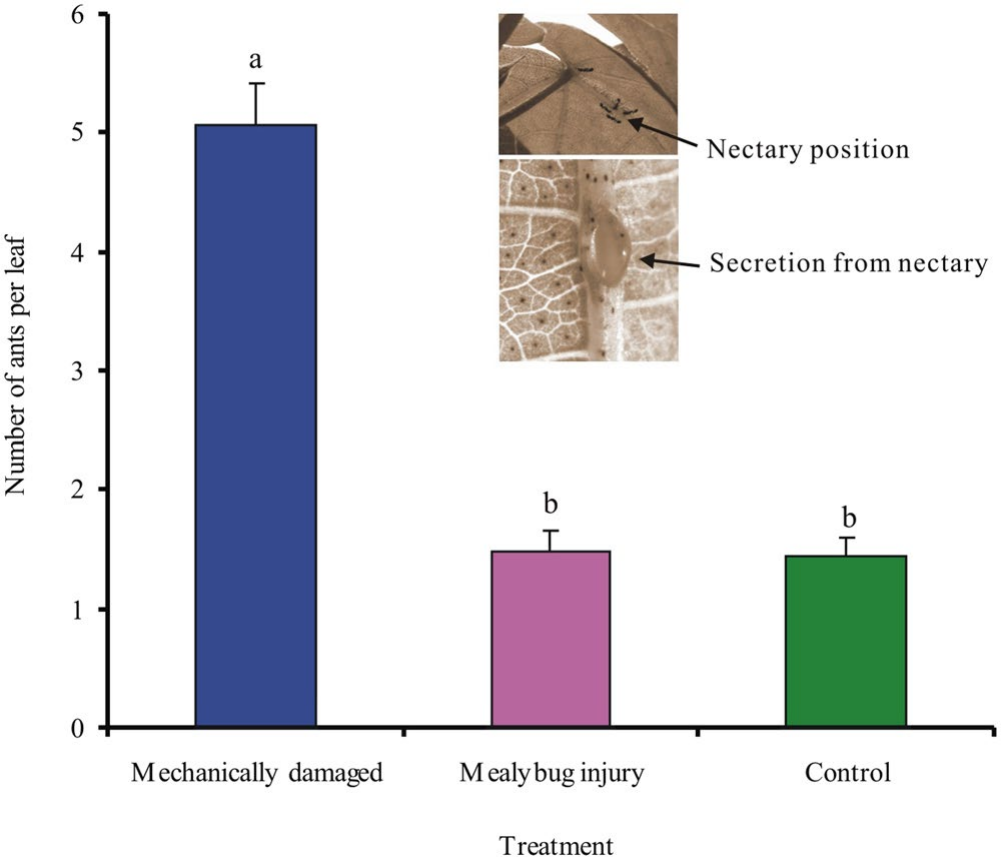
Numbers of ants near extrafloral nectaries located in the main veins on mechanically damaged, mealybug-infested, and control cotton leaves in experiment 3. The ants were counted 1 h after the experiment began. Each value is the mean (+SE) of 10 replications.

Ant recruitment was much greater and more rapid to mechanically damaged plants than to control or mealybug-damaged plants (Fig. 3). Ant number was significantly affected by treatment (*F*_2,189_ = 16.37, *P* < 0.001) and by the interaction between treatment and observation time (*F*_12,189_ = 41.97, *P* < 0.001) but not by observation time (*F*_6,189_ = 3.75, *P* = 0.025). After 1 h, the number of ants adjacent to the extrafloral nectaries on the main veins was significantly higher on the mechanically damaged plants than on the plants of the other treatments (*F* = 72.08, *df* = 29, *P* < 0.001) but did not differ between mealybug-damaged plants and control plants (Fig. 4).

### Responses of patrolling ants to plants with ant-tended mealybugs (experiment 4)

**Figure 5:**
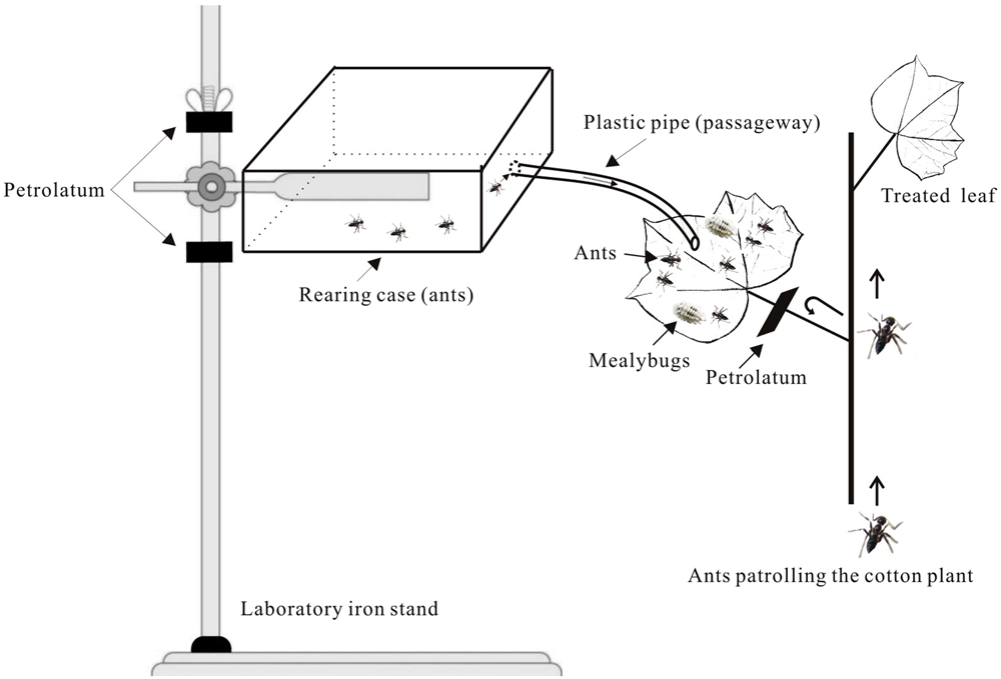
Diagram of the set-up used in experiment 4 to determine whether patrolling ants are attracted to plants with ant-tended mealybugs. The treatment illustrated is MA2, which is described in Fig. 6 and in the text.

**Figure 6:**
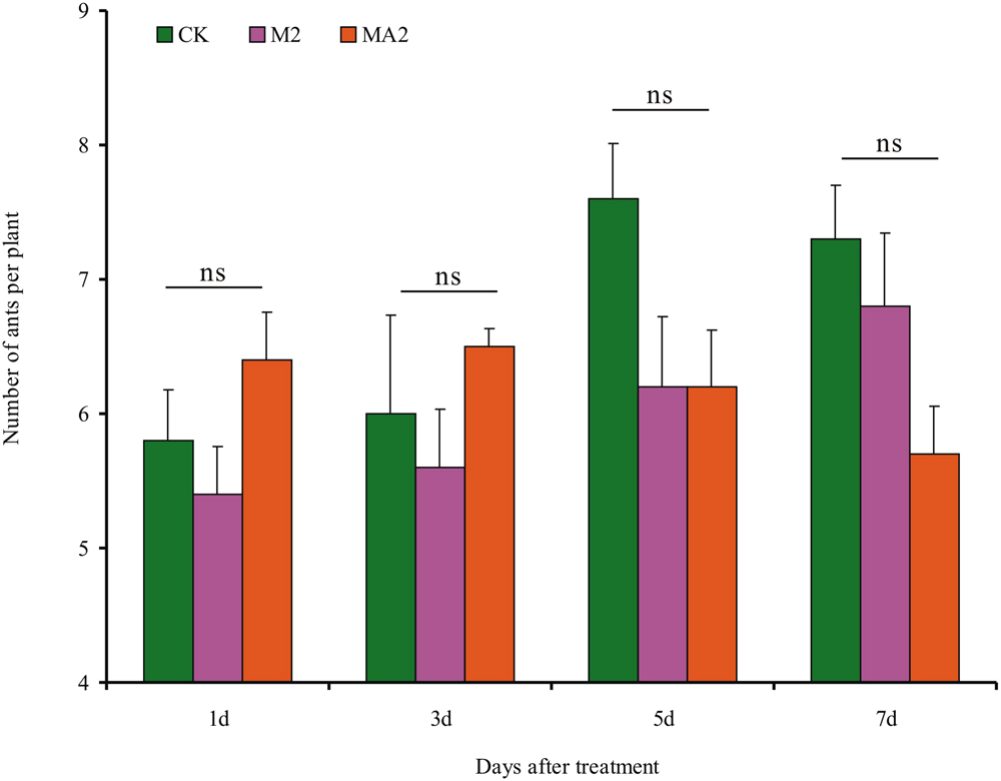
Responses of patrolling ants to plants with ant-tended mealybugs (experiment 4). Patrolling ants (ca. 200 ants) were present in all three treatments and could move up the plant to the petioles but could not move onto the leaf blades because the petioles were coated with petrolatum. In treatment MA2, ants in the rearing case (ca. 100 ants) had access to the leaf blades with mealybugs (60 individuals per plant). For treatment M2, leaf blades had mealybugs but no ants. In the control (CK), there were no ants or mealybugs on the leaf blades. Numbers of patrolling ants per plant were determined at 1, 3, 5, and 7 d after the mealybugs were placed on the leaves. Values are the means (+SE) of five replicates. The effects of treatment, observation time, and their interaction were not statistically significant (*P* > 0.05).

Experiment 4 determined whether patrolling ants were attracted to plants with ant-tended mealybugs (treatment MA2), with mealybugs alone (M2), or without mealybugs or ants. The experimental set-up is illustrated in Fig. 5. As indicated in the figure, the patrolling ants could move up the plant but could not contact mealybugs or tending ants because the petioles were coated with petrolatum. The number of patrolling ants per plant was determined at the indicated time intervals. The effects were not statistically significant for treatment (MA2, MA, and CK; *F*_2,60_ = 2.47, *P* = 0.093), observation time (*F*_3,60_ = 2.48, *P* = 0.070), or the interaction between treatment and observation time (*F*_6,60_ = 2.22, *P* = 0.053) (Fig. 6).

### Effects of the tending of mealybugs by ants on volatiles emitted from plants (experiment 5)

**Figure 7:**
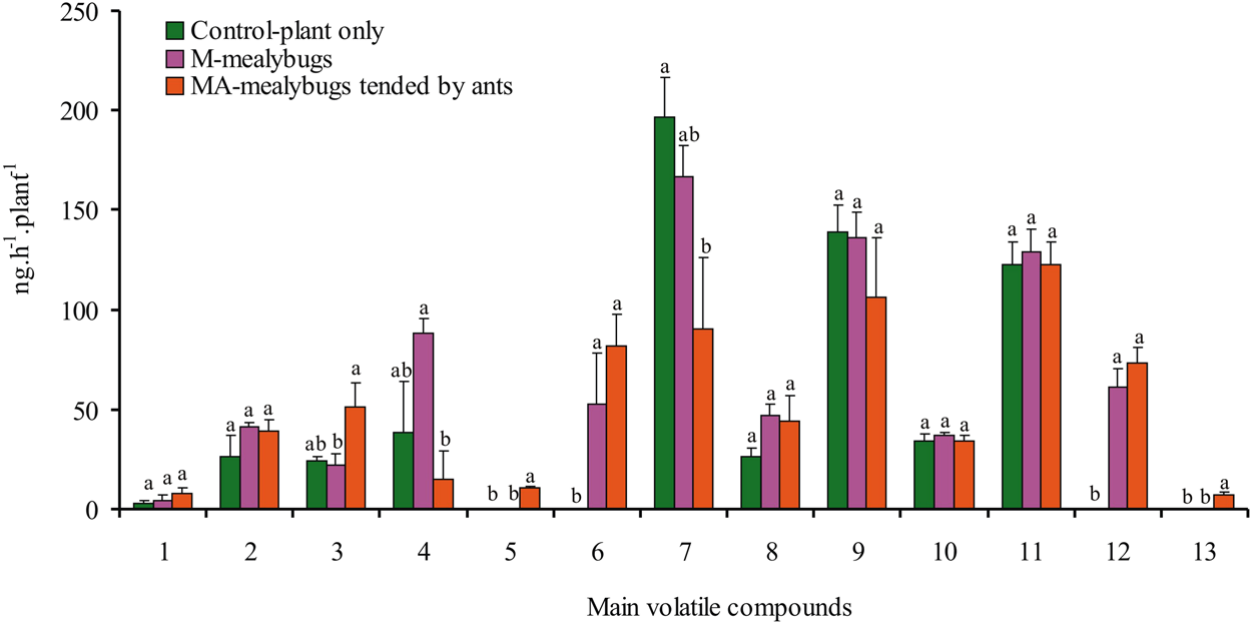
Means and standard error (ng.h^−1^.plant^−1^) of main compounds emitted by cotton infested with mealybugs (M) or infested with mealybugs tended by ants (MA) or infested no mealybugs (Control). Code 1~13 on axis X indicate the main volatile compounds, there are octanal, nonanal, ρ-cymene, methyl nicotinate (4), MeSA (5), β-curcumene (6), dodecane, decanal, tetradecane, laurylacetate, hexadecane, cedrol (12), and one unidentified compound (13), respecitively.

When volatiles were collected from cotton plants subjected to treatment (CK, M2, or MA2), a total of 13 major volatile compounds were detected; 9 in the CK (without mealybugs or ants), 11 in M2 (with 60 mealybugs per plant), and 13 in MA2 (with 60 ant-tended mealybugs per plant) (Fig. 7). Mealybugs caused the emission of cedrol whether or not the mealybugs were tended by ants (*F* = 23.16, *df* = 14, *P* < 0.001). When cotton plants were infested with mealybugs, methyl nicotinate and β-curcumene emissions tended to increase, whereas dodecane and p-cymene emissions tended to decrease, but the rate of emission did not significantly differ from that in the control. Relative to the control, MA2 significantly increased the emission of p-cymene (*F* = 3.92, *df* = 14, *P* = 0.049), β-curcumene (*F* = 12.70, *df* = 14, *P* = 0.0011), cedrol (*F* = 23.16, *df* = 14, *P* < 0.001), and one unidentified compound (*F* = 11.17, *df* = 14, *P* = 0.002), but significantly decreased the emission of methyl nicotinate (*F* = 3.92, *df* = 14, *P* = 0.049) and dodecane (*F* = 4.43, *df* = 14, *P* = 0.04). Methyl salicylate (MeSA) and one unidentified compound were emitted when ants tended mealybugs (MA2) but not when ants did not tend mealybugs (M2).

## Discussion

Previous studies have shown that honeydew-producing hemipterans may increase their sap feeding and their frequency of honeydew excretion^22^ and may also change the sugar composition of their honeydew^23^ in response to tending by ants. Because plant volatiles are closely related to the feeding status of pests^24^, it is reasonable to suspect that ants may indirectly change the emission of plant volatiles by tending honeydew-producing hemipterans^13^. The ecological consequences of mutualistic interactions between tending ants and honeydew-producing hemipterans differ among ant species, primarily owing to the differences among ant species in aggressiveness and territoriality^25^. For example, tending by a local ant, *Lasius grandis*, increased emission of α and β-pinene and sabinene by holm oak (*Quercus ilex*) saplings, whereas tending by an invasive ant, *Lasius neglectus*, decreased the emission of myrcene^13^. When the ghost ant tended the cotton mealybug in the current study, the emission of methyl nicotinate by cotton plants significantly decreased, and MeSA and one unidentified compound were detected; appeared in the emissions. MeSA and the one unidentified compound, in contrast, were not detected from control plants.

Plants emit significant quantities of MeSA, a volatile compound of green leaves, especially when they are stressed^26^, and MeSA is attractive to parasitic wasps^27^. We found that experienced *A. bambawalei* females showed a significant preference for the odor emitted by the cotton plants with ant-tended mealybugs. Determining whether this attraction was induced by MeSA or by other compounds (e.g., the emission of cedrol from mealybug-infested plants) will require further testing. Ant tending did not impede host searching by *A. bambawalei*. Our results indicate that, based on the sensing of herbivore-induced plant volatiles, *A. bambawalei* learns to more effectively locate host insects, which was not unexpected because the ability to learn in this manner is common in parasitoids^28^. Some results, however, were unexpected; attraction of *A. bambawalei* to plants, for example, tended to be greater when mealybugs were tended rather than not attended by ants. This was unexpected because the ants might attack *A. bambawalei* and cause the parasitoid to take evasive action^10^. However, one benefit we can expected is that, once female parasitoids learn to avoid or defuse ant interference during foraging^11^, their progeny will benefit from ant defense as they develop.

Ants are able to rapidly move to localized sites of leaf damage following the emission of chemical cues associated with herbivory^16^. In the present study, however, increase in cotton leaf damage over time following mealybug infestation did not increase ant recruitment (Fig. 2), whereas ant recruitment was greatly increase by mechanical damage that imitated the feeding of chewing insects (Fig. 3). We suspect that this difference may be explained by an increase in secretions from the extrafloral nectaries (EFNs) of cotton plants in response to mechanical damage. Certainly, we can not exclude the possibility that the volatiles that were induced by chewing insects, or that released from EFN itself^29, 30^, indicate to the ants that caterpillars are available as prey. Whether the marked increase in secretions from EFNs in response to mechanical damage, or the volatiles released from EFNs itself recruited ants remains to be investigated.

Previous studies showed that EFNs of many plants (at least 3941 species of plants in 745 genera and 108 families) produce a carbohydrate-rich substance (e.g., sugar and amino-acids) that is highly attractive to ants^31, 32^. The secretion of such compounds by EFNs functions as a reward for ants and is one of the multiple strategies that plants use to defend against herbivores^33, 34^. Infestation by mealybugs, however, did not apparently change EFN secretions from cotton plants, perhaps because phloem-feeders (like mealybugs) generally induce plant defenses by the salicylic acid (SA) pathway, and SA does not increase the secretion of EFNs^35, 36^. Furthermore, we recently found that the cotton mealybug can manipulate plants for its own benefit by modulating JA (jasmonic acid)-SA crosstalk in order to suppress induced defenses, i.e. the mealybug feeding enhanced SA accumulation, which suppressed the JA signaling pathway^37^. Heil reported that EFN secretion is commonly induced by wounding, likely owing to a JA-induced cell wall invertase, and is limited by phloem sucrose availability^31^. Therefore, the mealybug feeding may not change the secretion of EFNs, whether ants are present or absent, and this may explain why the ant was not attracted to mealybug-infested cotton plants in the current study. We speculate that foraging ants may not detect the changes in plant volatiles caused by mealybugs and therefore may not search for mealybugs proactively. Although the results might be explained by a failure of ants to detect changes in the quality of volatiles, they may also be explained by a failure of ants to detect low quantities of volatiles because emissions are lower with phloem feeding insects than with chewing insects^38^.

Although changes in plant volatiles following infestation of cotton plants by mealybugs did not result in ant recruitment, recruitment might occur when ants encounter mealybugs and receive honeydew as a reward^4^. We also suggest that the ant-tended mealybug association caused experienced *A. bambawalei* females to prefer damaged cotton plants to non-damaged plants. However, the response of *A. bambawalei* females to the danger posed by honeydew-collecting ant warrants further study, Additional research is also needed on the locating of hosts by *A. bambawalei* females at short distances as affected by volatiles generated by plants infested with ant-tended mealybugs.

The cotton mealybug is a highly polyphagous pest that damages >200 plant species from approximately 24 countries in tropical and subtropical regions of the world^39^. Since 2008, the mealybug has been rapidly spreading throughout South China^18^. Our laboratory data indicate that the mutualism between the mealybug and the ant on cotton plants affects the behavior of *A. bambawalei* (but not that of patrolling the ant) via changes in the emission of volatile compounds from the plants.

## Methods

### Plants and insects

Cotton plants, *Gossypium hirsutum* (Malvales: Malvaceae; cv Zhemian-607), were grown in plastic pots (11 cm diameter, 9 cm height) containing sterilized turf soil. The potted plants were placed in a greenhouse (30 ± 2 °C, 70 ± 5% RH) at the Flower Research and Development Center, Zhejiang Academy of Agricultural Sciences (30°18′75″ N; 120°28′60″ E), Hangzhou, China. Plants were fertilized with plant nutrient solution (18-2-10, N-P-K) and were watered regularly. Plants were used for experiments when they had nine to ten fully expanded true leaves (ca. 35 cm height). For experiments, the soil in the pots was covered with a freshness protection package and aluminum foil (25 × 35 cm, Cleanwrap Co., Ltd., Seoul, Korea) to prevent ants from establishing a new nest in the soil. Preliminary tests showed that this treatment had little effect on plant growth or volatile emission.

The cotton mealybug *P. solenopsis* was reared on cotton plants in a climate chamber (27–30 °C, 50–70% RH, and a L16: D8 photoperiod). Based on previous studies^4^, third-instar nymphs of *P. solenopsis* were used in the experiments because of their relatively high survival rate and suitable duration of development.

The ghost ant *T. bicarinatum* is widely distributed in greenhouses in China, where its tend to various species of honeydew-producing insects^40^ and feeds on the extrafloral nectaries of plants. The methods used to collect and rear *T. bicarinatum* similar to those of Banks *et al*.^41^. Colonies were reared in square plastic boxes (42 cm length × 28 cm width × 18 cm height, coated with Fluon® on the inside walls to prevent escape) that were kept in the same climate chamber as the mealybugs. Each colony contained 150–200 workers plus two queens. The ants were starved for 48 h before the start of the experiments.

The parasitoid wasp *A. bambawalei* is a koinobiont nymphal endoparasitoid of the mealybug, and the third-instar mealybug is the preferred stage for its development^42^. A laboratory colony was established with the mealybug as the hosts in May 2012 and was maintained in a growth chamber at 30 ± 2 °C with a 14 L: 10D photoperiod and 75 ± 5% RH. The sex of *A. bambawalei* individuals was visually determined based on body size and antennae morphology.

### Attraction of inexperienced and experienced wasps to plants with and without mealybugs that were tended or were not tended by ants (experiment 1)

*Aenasius bambawalei* female adults were divided into two groups: inexperienced and experienced. Inexperienced individuals were not exposed to cotton plant odors before the experiment began, and they were tested within 24 h after emergence from mealybug mummies. The inexperienced individuals were allowed to mate and were provided with ample water and honey but remained isolated from cotton plant odors. The experienced individuals were exposed to cotton plant odors 48 h after emergence (i.e., they had a learning experience), but like inexperienced individuals, the experienced individuals had no oviposition experience. For the learning experience, the cotton plants had been infested with mealybugs alone (30, 60, or 90 per plant); were infested with mealybugs (30, 60, or 90 per plant) and tending ants; or were not infested with mealybugs or ants. These treated plants were obtained as described in experiment 2, except that the petioles were not covered with petrolatum.

The plant response to honeydew-producing insects usually consists of the systemic production of volatiles for up 24 h after the honeydew-producing insects are removed from the plant^43^. A glass Y-tube olfactometer was used to determine the preference of inexperienced and experienced *A. bambawalei* female adults for pairs of different volatile sources. The Y-tube olfactometer bioassay followed the protocols described by Edwards *et al*.^15^. An air pump (model “ZC-Q”; Zhejiang Hengda Instrument and Meter Co., Ltd., Hangzhou, China) pushed air through glass U-tube filters containing activated carbon and a molecular sieve (Sigma-Aldrich, Saint Louis, USA) to purify the air. Air then passed through Teflon tubing into a flow meter (model LZB-3WB; KEDE Electric, Co., Ltd., Changde, China) set at 250 ml/min and then into two glass cylinders (23 cm diameter, 40 cm height) like those described by Zhang *et al*.^44^. Each glass cylinder contained two test plants. Air that left one glass cylinder moved through Teflon tubing and into one arm of the olfactometer, and air that left the other glass cylinder moved through Teflon tubing and into a second arm of the olfactometer. The arms of the olfactometer were 15 cm long (2.5 cm internal diameter) at a 60° angle, whereas the third arm was 16 cm long with the same internal diameter. All tests were performed at 27 ± 2 °C. The Y-tube was lit from above by two 30 W fluorescent lamps (light intensity ca. 3.5 klx). The system was run for 15 min before the experiment.

A trial began with the release of one *A. bambawalei* female into the third arm of the Y-tube. Each female was given 5 min to choose. A “no choice” was recorded when the female remained inactive, and a choice for one of the two odor sources was recorded when the female moved >5 cm into one of the arms and remained for at least 15 s. The position of the treatments was swapped between the arms of the Y-tube every four replicates. Between trials, all glassware was washed in a detergent, rinsed with 95% ethanol, and distilled water and then baked at 200 °C for 2 h to remove any volatile chemicals adhering to the glass.

Experiment 1 included five odor comparisons: Control (CK) vs. 60 mealybugs per plant (M2); CK vs. 60 mealybugs per plant + tending ants (MA2); M2 vs. MA2; 30 mealybugs per plant (M1) vs. 30 mealybugs per plant + tending ants (MA1); and 90 mealybugs per plant (M3) vs. 90 mealybugs per plant + tending ants (MA3).

### Ant responses to mealybug infestation (experiment 2)

To determine whether foraging ants are attracted to leaves damaged by mealybugs based on the release of volatile chemicals, we monitored ant numbers on plants with and without mealybugs. A cotton plant with 30, 60, or 90 mealybugs per leaf (one leaf per plant) was placed on a round iron rack (7 cm height × 13.5 cm diameter) that set in a box contain ca. 200 workers plus two queens. The rack served as a bridge between the plant and the ants. Petrolatum applied to the petioles of the leaves prevented the ants from contacting the mealybugs. Preliminary tests showed that petrolatum did not affect ant foraging. Beginning at 24 h after the mealybugs were added, ant numbers on cotton plants were recorded at 10:30 a.m. and 2:30 p.m. each day (ants were counted for ca. 1 min) for 5 consecutive days; other details were similar to those described by Stanley *et al*.^3^. Each treatment was replicated 10 times. The experiment was conducted in a temperature-controlled clean room (28 ± 2 °C, 60–70% RH, and a 14 L: 10D photoperiod).

### Ant responses to mechanical damage (Experiment 3)

This experiment determined whether foraging ants would rapidly move to mechanically damaged or mealybug-infested cotton leaves. Cotton plants without mealybugs (CK), with 60 mealybugs per plant (M2), and with mechanical damage were used. The mechanical damage was similar to that caused by a chewing insect; such damage can induce rapid ant recruitment^16^. A hole punch (0.4 cm diameter) was used to create two holes in each leaf of two randomly selected leaves per plant. The treated leaves were also isolated by petrolatum. Mealybug-injured plants of the M2 treatment were undisturbed for 24 h before each pot was placed on a rack sitting in a box containing ca. 200 ants as described for experiment 2. The ants could move to the plants but could not contact the leaves because the petioles were coated with petrolatum. Ant numbers per plant were recorded after 5 min, 15 min, and 30 min and 1 h, 3 h, 6, and 24 h. Ants near extrafloral nectaries were also recorded after 1 h. Each treatment was replicated 10 times.

### Responses of patrolling ants to plants with ant-tended mealybugs (experiment 4)

To test whether ant-tended mealybugs attract patrolling ants, we used the equipment shown in Fig. 5. The plant treatments were CK, M2, and MA2. Patrolling ants (ca. 200 ants), which were present in all three treatments, could move up the plant to the petioles but could not move onto the leaf blades because the petioles were coated with petrolatum. In treatment MA2, ants in the rearing case (ca. 100 ants) had access to the leaf blades with mealybugs (60 individuals per leaf). For treatment M2, leaf blades had mealybugs but no ants. In the control (CK), there were no ants or mealybugs on the plants. Patrolling ants on plants were counted at 10:30 a.m. and 2:30 p.m. after 1, 3, 5, and 7 d. Each treatment was replicated five times.

### Effects of the tending of mealybugs by ants on volatiles emitted from plants (experiment 5)

In this experiment, we tested the hypothesis that ants indirectly change the composition and content of plant volatiles by tending to mealybugs. The following three treatments were compared: CK, M2, and MA2. These treatments were applied as described for experiment 2. Volatile compounds were collected from two cotton plants from the same treatment.

The system used for collection of volatiles was identical to that described in experiment 1, except for the glass Y-tube. The rate of airflow was 300 ml/min. To create a laminar flow, the air was forced through a glass frit at the top of the cylinder. The cylinder had a 25-mm vertical female ground-glass connector for a collection trap 4 cm above the bottom (see Zhang *et al*.^44^). The trap was a glass tube (15 cm length, 5 mm diameter) that contained 60 mg of 80/100 mesh Porapak-Q (Waters Corporation; Milford, MA). The air passing over the plants was pulled through the Porapak-Q adsorbent and vented out. Collection began at 9:30 a.m. and continued for 2 h in each trial, and 300 ng of nonyl acetate (Sigma) was added as an internal standard. After each collection, the trap was rinsed with 500 µl of methylene dichloride. Volatiles were also collected from a blank cylinder to verify that system was clean. Each treatment was replicated five times. Samples were stored at −20 °C until further analyzed.

The compounds in each sample were identified by GC-MS analysis with an Agilent 5973 B (Santa Clara, CA, USA) mass selective detector coupled with an Agilent 6890 N network GC system equipped with a quartz capillary column (HP-5 MS, 30 m-0.25 mm i.d., 0.25 µm film thickness; J&W Scientific, Palo Alto, CA, USA). For each sample, 0.5 µl was analyzed on the column in splitless mode with an injection port temperature of 250 °C. Following injection, the column temperature was maintained at 40 °C for 3 min, increased to 200 °C at 6 °C/min, and held at 200 °C for 3 min. Helium was used as the carrier gas at 1 ml/min at a constant flow rate. By crosschecking with the mass spectrum fragment database, candidate bioactive compounds were identified, and final identification was confirmed with standard chemical spectrum patterns. The concentration of total chemical blends was calculated using a standard chemical curve developed from gradient GC-MS spectra.

### Statistical analysis

*Chi-square tests* were used to compare wasp attraction of each pair of volatile sources in experiment 1. We calculated the average number of ants on the plants at 10:30 a.m. and 2:30 p.m. for each day and then used repeated-measures ANOVA to compare ant numbers between mealybug-infested intensity (between-subject) and between observation days (within-subject) in experiment 2; when ANOVA were significant, performed Duncan’s tests were used for multiple comparisons. Similarly, the data on ant number were also compared among different treatments at different observation time points with repeated-measures ANOVA in experiment 4. A one-way ANOVA was used to compare treatment effects on ant numbers near extrafloral nectaries at 1 h after the start of the experiment in experiment 3. For analysis of volatiles emitted in experiment 5, one-way ANOVAs were used to compare the differences between treatments, and multiple comparisons were performed with Duncan’s test. All statistical analyses were conducted using the SPSS 14.0 statistical software package (SPSS Inc., Chicago, IL, USA).
